# Prevalence and treatment of asymptomatic bacteriuria at academic and critical-access hospitals—Opportunities for stewardship efforts

**DOI:** 10.1017/ice.2022.143

**Published:** 2023-06

**Authors:** Funnce Liu, Barbara MacDonald, Rupali Jain, Chloe Bryson-Cahn, Natalia Martinez-Paz, John B. Lynch, Paul S. Pottinger, Jeannie D. Chan, Zahra Kassamali Escobar

**Affiliations:** 1 Department of Pharmacy, University of Washington Medical Center, Montlake, Seattle, Washington; 2 School of Pharmacy, University of Washington, Seattle, Washington; 3 Healthcare Management Alternatives, Inc, Vashon, Washington; 4 Tele-Antimicrobial Stewardship Program, University of Washington, Seattle, Washington; 5 Department of Pharmacy, Harborview Medical Center, Seattle, Washington; 6 Department of Medicine, Division of Allergy & Infectious Diseases, School of Medicine, University of Washington, Seattle, Washington; 7 Department of Pharmacy, UW Medicine Valley Medical Center, Renton, Washington

## Abstract

Asymptomatic bacteriuria (ASB) is common among hospitalized patients and often leads to inappropriate antimicrobial use. Data from critical-access hospitals are underrepresented. To target antimicrobial stewardship efforts, we measured the point prevalence of ASB and detected a high frequency of ASB overtreatment across academic, community, and critical-access hospitals.

Asymptomatic bacteriuria (ASB), the presence of bacteria in the urine without signs or symptoms of urinary tract infection (UTI), is a common clinical finding, particularly in the elderly and in women.^1^ Most patients with ASB derive no benefit from treatment, and evidence suggests that treatment does not prevent progression to symptomatic UTI, complications, or death.^1^ Nevertheless, antibiotics are often prescribed for ASB. Notably, the indiscriminate use of antibiotics not only contributes to the emergence of antibiotic resistance and *Clostridioides difficile* infection but also causes harmful side effects and may increase the risk of subsequent symptomatic UTI.^1^ Overtreatment of ASB has therefore been identified as a key opportunity to reduce unnecessary antibiotic use and to minimize adverse effects. A recent meta-analysis reported a pooled prevalence of 45% for ASB treatment among mostly hospitalized patients at academic medical centers,^2^ but the prevalence has not been well characterized across the spectrum of care. Critical-access hospitals (CAHs) are underrepresented in the literature. To target antimicrobial stewardship efforts in our region, we assessed the point prevalence of ASB and the frequency of ASB treatment across academic, community, and critical-access hospitals.

## Methods

In this multisite study, we included patients aged ≥18 years with a positive urine culture showing ≥100,000 colony-forming units (CFU)/mL of 1 or more bacteria. Pregnant patients were excluded. The study included four University of Washington (UW) academic and community hospitals (281–529 beds) and 5 CAHs (≤25 beds) participating in the UW Tele-Antimicrobial Stewardship Program. Each site collected data from up to 50 patients at their own discretion during a 1-month window between November 2020 and January 2021 and submitted deidentified results into REDCap, an electronic data management program. Data were analyzed with descriptive statistics using the Fisher exact test. A 2-sided *P* value <.05 was considered statistically significant. The study was approved by the University of Washington institutional review board and a requirement for informed consent was waived.

ASB was defined as a positive urine culture without symptoms of UTI using the National Hospital Safety Network definition.^3^ Because diagnostic criteria are based on the subjective symptoms of UTI, the ability of patients to answer questions was essential to optimally adjudicating the appropriateness of antimicrobial treatment. All medical notes within the infection window period, defined as the date of urine collection as well as 3 days before and 3 days after, were reviewed for evidence of patients answering symptom-based questions. If reviewers were unable to discern this information, documentation of the patient being awake and oriented to person, place, and time (ie, A&O×3) was used as a surrogate marker for the ability to answer questions. However, to reflect real-world practice, patients unable to answer questions were still included in the final analysis.

Treatment of ASB was defined by documentation of a specific antibiotic treatment for bacteriuria. Use of antibiotics for other indications was determined from patient care notes or indications associated with the antibiotic orders.

## Results

In total, 275 patients were identified: 191 (69.5%) from the UW Medicine system and 84 (30.5%) from CAHs. Among them, 75% were female, and the median age was 64 years (Table 1). An indwelling urinary catheter was present in 60 patients (21.8%). Urine cultures were predominately collected in the emergency department (ED; 76.0%). Moreover, 225 patients (81.8%) were able to answer symptom-based questions. Symptoms of UTI were documented in 92 patients (33.5%), and 11.3% had a temperature >38°C.

**Table 1.** Baseline Characteristics of Patients With Positive Urine Culture

Variable	Overall (N=275), No. (%)	Overall (N=275)		Asymptomatic Bacteriuria (N=161)	
		Academic & Community Hospitals (N=191), No. (%)	Critical-Access Hospitals (N=84), No. (%)	Treated (N=112), No. (%)	Not Treated (N=49), No. (%)
Age, median y [IQR]	64 [46–77]	63 [46.5–74]	68 [46–79.5]	67 [57–82]	59 [36–72]
Sex, female	207 (75.3)	144 (75.4)	63 (75.0)	82 (73.2)	39 (79.6)
Urinary catheter^a^	60 (21.8)	45 (23.6)	15 (17.9)	31 (27.7)	6 (12.2)
**Location of urine culture collection**					
ED then discharged	124 (45.1)	72 (37.7)	52 (61.9)	40 (35.7)	24 (49.0)
ED then admitted to hospital	85 (30.9)	58 (30.4)	27 (32.1)	43 (38.4)	11 (22.4)
Medical wards	46 (16.7)	44 (23.0)	2 (2.4)	21 (18.8)	8 (16.3)
Intensive care units	8 (2.9)	8 (4.2)	0 (0.0)	5 (4.5)	0 (0.0)
Rehabilitation or long-term care	6 (2.2)	6 (3.1)	0 (0.0)	3 (2.7)	2 (4.1)
Other	6 (2.2)	3 (1.6)	3 (3.6)	0 (0.0)	4 (8.2)
**Ability to answer questions**					
Yes	225 (81.8)	149 (78.0)	76 (90.5)	78 (69.6)	43 (87.8)
No	50 (18.2)	42 (22.0)	8 (9.5)	34 (30.4)	6 (12.2)
Fever (>38°C)	31 (11.3)	20 (10.5)	11 (13.1)	…	…
Documented urinary symptoms	92 (33.5)	59 (30.9)	33 (39.3)	…	…
Suspected or confirmed coinfection	58 (21.1)	44 (23.0)	14 (16.7)	35 (31.3)	1 (2.0)

Note. ED, emergency department; IQR, interquartile range.

a The presence of a Foley catheter was counted if it was present either on the day of or day before the urine culture was obtained.

**Fig. 1.**
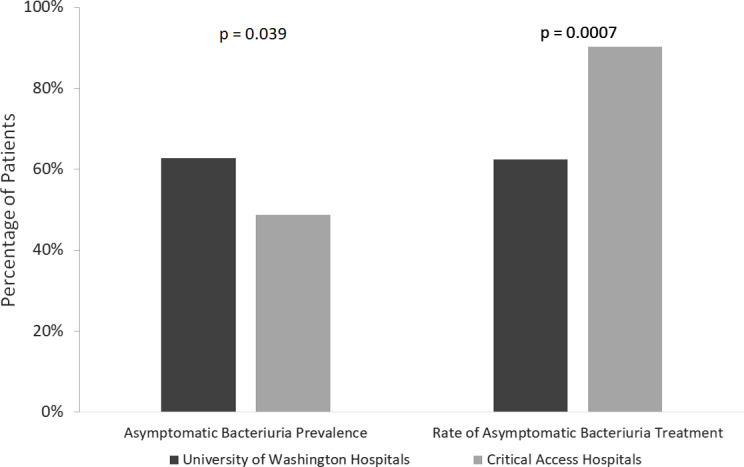
Asymptomatic bacteriuria prevalence and proportion treated at University of Washington hospitals and critical-access hospitals.

ASB was identified in 161 (58.5%) of 275 patients: 120 (62.8%) of 191 patients at UW hospitals and 41 (48.8%) of 84 patients at CAHs (*P* = .039). Among patients with ASB, antibiotics were prescribed in 112 (69.6%) of 161 patients: 75 (62.5%) of 120 patients at UW hospitals and 37 (90.2%) of 41 patients at CAHs (*P* = .0007) (Fig. 1). When patients with concern for concomitant infections from a nonurinary source were excluded, antibiotics were still prescribed for ASB in 77 patients (47.8%). The median treatment duration of ASB was 6.5 days; ceftriaxone (26.8%) was the most prescribed antibiotic.

Among patients treated for ASB, compared to patients not being treated for ASB, a higher proportion had indwelling urinary catheters (27.7% vs. 12.2%; *P* = .041), concerns for a concomitant infection (31.3% vs 2.0%; *P* = .0001), and inability to answer questions (30.4% vs 12.2%; *P* = .017).

## Discussion

In this study of 275 patients, we observed a 58.5% prevalence of ASB, ranging between 48.8% at CAHs and 62.8% at academic and community hospitals, respectively. The frequency of ASB treatment was 69.6%, varying between 62.5% at academic and community hospitals and 90.2% at CAHs, respectively. ASB is highly prevalent, and inappropriate treatment of ASB is common at both large and critical-access hospitals, a finding consistent with previously published studies.^2,4,5^ Although the prevalence of ASB was lower in CAHs, suggesting more selective criteria for sending urine culture, the high rate of antibiotic prescribing reveals opportunities for targeted stewardship interventions, specifically establishing diagnostic and treatment criteria and shortening treatment durations as recommended by the Centers for Disease Control and Prevention.^6^

In our study, urine cultures were primarily ordered in the ED, which may be a high-yield area for stewardship interventions. Shallcross et al^7^ reported that >60% of patients treated for UTI syndromes in the ED lacked clinical evidence of infection, yet antibiotics were continued in >75% of patients. Their finding is supported by another study in which failure to re-evaluate the need for antibiotics initiated in the ED for presumed UTI led to inappropriate continuation of therapy in hospitalized patients.^8^

The median treatment duration for ASB was 6.5 days in our study, which represents another stewardship opportunity. Guidelines for UTI in women recommend 3–5 days as the shortest effective therapy.^9^ Furthermore, each day of antibiotic therapy is associated with 4% increased odds of experiencing an adverse event.^10^ Even if treatment of ASB cannot be completely avoided, shortening duration of therapy may be a compromise to reduce unnecessary antibiotic exposure and potential harm.

Although data to guide interventions in CAHs are limited, we must consider that CAHs experience unique challenges related to their size and remote locations. These challenges include access to and turnaround time with microbiology data; many hospitals outsource testing to a reference or commercial laboratory, which may be far away. Healthcare personnel often include traveling locums, which may impede a consistent practice environment or shared decision making between providers who order and interpret a urine culture.

The strength of our study is the chart review of individual patient records to ascertain and adjudicate ASB cases and treatment, therefore minimizing the potential for misclassification. Our study had several limitations. The sample size was small due to lower patient volume in critical-access hospitals and by a 1-time timeframe for our data collection. Additionally, the CAHs that participated in this review were self-selected and do not necessarily represent the full spectrum of ASB management among CAHs. To our knowledge, this is the first study that has attempted to characterize the prevalence and treatment of ASB in less-studied CAH settings.

In conclusion, our findings illustrate the widespread overtreatment of ASB and highlighted areas for targeted interventions. However, we cannot effectively address the downstream effects of the overtreatment of ASB without directing interventions upstream to reduce the detection and overdiagnosis of ASB in the first place.^11^ Although the excessive ordering of urine culture is a strong driving factor that perpetuates the inappropriate treatment of ASB, it is also important to recognize that CAHs may face unique barriers. We call for not only of diagnostic and antimicrobial stewardship to promote prudent use of urine testing and thoughtful antibiotic prescribing but also pinpointing CAH-specific challenges as a patient safety and quality improvement initiative.
